# Mouth Infections and Their Systemic Effect

**Published:** 1916-12

**Authors:** Arthur H. Merritt

**Affiliations:** New York


### Mouth Infections and Their Systemic Effect.

BY ARTHUR H. MERRITT, D. D. S., NEW YORK.

The discovery that chronic focal infections might be, and not infrequently were, the cause of more or less serious systemic diseases, placed a new responsibility upon those who undertook the treatment of human ills.

The importance of such infections, and the necessity for their elimination, became apparent when it was found that obscure and painless infections might produce secondary lesions in remote parts of the body, the seriousness of which were wholly out of proportion to the exciting focus. Thus a new light was thrown upon the etiology and treatment of certain diseases, the nature of which had hitherto been but little understood. The mouth, more often than any other part of the body, is the site of such infections, the frequency of which was wholly unsuspected before the Roentgen ray came into general use. Such being the case, no physical examination is complete which does not include a careful scrutiny of the mouth. This must be more than the casual examination for decayed teeth made by the physician; more even than the perfunctory inspection too often made by the dentist. Nor does the physician's responsibility cease by advising the patient to "Go and see your dentist," as is so often done. Since he is not, with few exceptions, qualified to make these examinations, it is his duty to accompany the patient to the dentist, and with him go over the case, that the presence or absence of such infections be ascertained beyond question, and if found, see to it that they are eliminated. To do less than this is to do less than one's duty. Since this is of first importance in every case of suspected infection, it is the purpose of this article to outline very briefly some of the more common mouth infections, that the physician may the more intelligently advise his patient in their treatment. Also to emphasize the importance of discrimination in the selection of a dental consultant, who must be not only a skilled diagnostician within his specialty, but also skilled in dental radiology, and radiographic interpretation, since these latter play so large a part in the successful treatment of these cases.

MOUTH HYGIENE.

The first thing to be considered in an examination of the mouth is its hygiene. There are mouths so filthy, that though there be no lesions, they are a menace to the patient's health, harboring, as they do, vast numbers of organisms which are constantly finding their way into the gastro-intestinal tract. Such conditions, however, are usually associated with a chronic gingivitis, permitting direct germ absorption. This should be corrected by proper treatment and patients instructed in the daily care of their mouths.

CROWN AND BRIDGEWORK.

Much crown and bridgework is constructed without due regard to the health and hygiene of the mouth. As a rule, fixed bridgework is unsanitary and should, therefore, be carefully examined. When so constructed as to interfere with the proper cleansing of the mouth, it should be removed. A careful examination should also be made of the gum margins around crowned teeth. Not infrequently, as a result of trauma, chronic infection is present, accompanied by a slight, though constant discharge of pus as shown by slight digital pressure. No such condition should be allowed to exist. Removal of crowns will usually result in prompt cure.

IMPACTED TEETH.

Still another source of infection, and one often unsuspected, is impacted teeth, usually third molars or so-called "wisdom teeth." It frequently happens that such teeth through lack of room are prevented from taking their normal place in the arch, resulting in deep crypts around the partially erupted tooth, which becomes chronically infected, with possible acute exacerbations. More often, however, they exist for years without any local symptoms, discharging pus from around the overlapping gum tissue, a constant menace to the health an well being of the patient. In not a few instances they are the primary source of tonsillar infections. All such teeth should be removed since there can be no possible excuse for retaining them save the natural desire to escape the disagreeable operation of extraction.

### DENTAL ABSCESSES.

One of the most common infections, and one requiring great care in diagnosis and treatment, is that which takes place in the periapical tissues,—the dental abscess. They are caused by non-vital teeth and are of two types, the fistulous and non-fistulous, or so-called blind abscess. They differ little in their pathology or bacteriology. With the development of caries in a tooth it is only a question of time unless arrested when the pulp will become involved, resulting in its death. This, in turn, will be followed by infection, the products of which find their way through the foramen, causing infection of the adjacent tissues. Devitalization of the pulp may and frequently does occur in teeth that are filled or crowned. Acute symptoms such as pain, swelling, temperature, loss of appetite, etc., may follow the infection of the periapical tissues. More often the infection becomes chronic without local symptoms. Radiographically, this infection will be represented by a rarefaction varying from the slightest departure from normal, to complete bone loss, with cavity formation in the body of the bone of considerable size. Such infections may exist for years without any local symptoms, and in case of blind abscess without any clinical evidence of their presence. Every non-vital tooth should, therefore, be examined radiographically in suspected cases, as in no other way can one be certain of the presence or absence of infection. Such teeth must always be regarded as potentially dangerous, though it is possible to reduce this to a safe minimum by proper treatment. This should consist in the removal of the gangrenous tooth pulp, disinfection of the root-canals and filling same to end under aseptic precautions. The organisms associated with these infections is a streptococcus, usually the viridans. In all cases of secondary infections, where the dental abscess is believed to be the primary focus, it is desirable to obtain an uncontaminated culture before it is obliterated, with a view to making an autogenous vaccine later should the secondary lesions not clear up by removal of the primary focus. In cases where the infection is such as to make extraction necessary, it is important to follow it up by a curettage of the infected area. In many instances the tooth may be saved by amputation of the root end, and curettment of the necrotic cavity. These are all problems which must be considered, the proper solution of which will have to be determined in each individual case.

PYORRHEA ALVEOLARIS.

Pyorrhea alveolaris is a disease involving the bony support of the teeth. It begins at the gum margins, and unless arrested, progresses by slow stages until the teeth are exfoliated. Its causes vary with individual cases, heredity, occlusal trauma, bad hygiene, etc., being among them. The usual symptoms are, loosening of the teeth, recession of the gums, which may or may not be inflamed, discharge of pus around the necks of the teeth on pressure, and possible elongation or separation of the teeth. It is essentially a disease of adult life, being most common after thirty years of age, is of slow progress, often existing in the mouth throughout the whole of adult life. In its early stages it is not painful and is usually unnoticed. Its most common characteristic is pus discharge, the amount of which will depend upon the number of teeth involved, and the stage to which the disease has progressed. When the pyorrheal pockets are deep, there is more or less direct absorption of living organisms into the blood stream. Most of the discharge, however, finds its way into the digestive tract. So far as is known, there is no specific organism which causes pyorrhea. The cocci group is represented by many strains, spirochaetes are exceedingly abundant, and certain types of anaerobes, notably Vincent's fusiform bacilli, are always present. The ameba is also present. There is no distinctive bacterial flora in pyorrhea. Qualitatively, it does not differ from that of the normal mouth. Quantitatively, it is always greatly increased, the more filthy the mouth, the greater the bacterial growth. The infection is probably always secondary, giving rise to certain of its symptoms, but sustaining no causal relation to it.

THE AMEBA IN PYORRHEA.

The ameba is probably always present in typical cases of pyorrhea, as it is in mouths that are unsanitary. It is also frequently present in clean and healthy mouths, though as a rule the more filthy the mouth, the more likely is it to be present. There is no evidence that it has anything to do with the cause of pyorrhea, all statements to the contrary, notwithstanding. It, doubtless, in common with the bacteria, finds expression in certain of its symptoms, but sustains no other relation to it. Unfortunately, many have been taught to believe that it was the causative agent, and that its destruction by emetin would effect a cure, with the result that many not familiar with the pathology and bacteriology of pyorrhea have been deceived by these specious claims. In consequence, physicians with no clinical knowledge of its treatment have given their patients emetin in the belief that thereby they were curing the disease. The fact that in some cases there is a temporary improvement following its administration encouraged them in this belief.

### TREATMENT OF PYORRHEA.

The only rational treatment, in fact, the only one that can possibly succeed, consists in the removal of all foreign matter from around the teeth, the careful curettage of all pockets, the planing of the root surface to insure complete removal of the necrotic peridental membrane, the correction of the occlusion, and the establishment and maintenance of normal hygiene. Autogenous or stock vaccines have no place in its treatment, though these, unfortunately, are being recommended by a few despite the fact that not one of them has ever been able to show that the organisms used sustained any casual relationship to the disease, and no two agree as to what organisms should be employed. In its early stages, it is easily and permanently cured. The more advanced cases are curable so long as there remains sufficient bone surrounding the root to support the tooth under the stress of mastication. In all cases where the disease has reached an incurable stage, or where for any reason treatment cannot be had, the teeth should be extracted, since artificial teeth in a healthy mouth are preferable to natural teeth hopelessly involved in a disease so potent for evil as is pyorrhea alveolaris.

### SYSTEMIC EFFECTS OF MOUTH INFECTIONS.

It is not purposed here to do more than call attention very briefly to some of the systemic effects of mouth infections, as the physician is usually best qualified to judge of these. It may, however, not be out of place to direct attention to some of the more obvious effects if for no other reason than to increase interest in this subject. It should be remembered, moreover, that the systemic effects of long continued absorption from some chronic focus, are not always uniform, nor are they always clearly defined. In some cases they seem to have no effect at all; certainly none that can be determined. In others, cause and effect are unmistakable, while still others prejudice, though they may not actually have caused, or have been the sole cause, of the systemic disturbance. This latter should be kept clearly in mind, that though they may not have caused the existing disturbance, they unfavorably influence the prognosis, and should, therefore, be eliminated. This should always be insisted upon.

### MALAISE.

A common expression of mouth sepsis is malaise. This may be associated with other symptoms, but it frequently exists by itself as a feeling of great lassitude which at times amounts almost to prostration. More often it obtains as a "tired feeling," which may vary in intensity from day to day, or be altogether absent certain hours of the day.

### ARTHRITIS.

There can be no doubt that arthritis is frequently the direct result of chronic mouth infections. The growth of mouth organisms under lowered oxygen tension such as prevails in dental abscesses or deep pyorrheal pockets, favors their acquirement of affinities for other tissues, such as the gastric mucosa, endocardium cartilages, etc. In every case the mouth should be carefully scrutinized, since it more often than any other part of the body is subject to conditions favoring secondary infections. When the relationship is fairly obvious, and the arthritis is of long standing, the desirability of obtaining a culture for vaccines before the focus has been eliminated should be considered. It is surprising, however, how often the arthritic condition clears up after the removal of the exciting focus.

### DIGESTIVE DISORDERS.

There has long existed a vague impression that in some way the digestive apparatus might be unfavorably influenced by the condition of the mouth. But not until recently has there been any realization of how intimate was this relationship or that the disturbance might take place indirectly through the vascular system. Such relationship exists and many cases of digestive disorders have for their primary cause a diseased and unsanitary mouth. No form of treatment should omit to take this into consideration.

### ENDOCARDITIS.

It has long been known that generalized infections such as scarlet fever, or acute localized infections such as tonsilitis, might produce endocarditis as a sequella, but that the same effect might be brought about by a chronic and less generalized infection, was less certain. That such relationship does exist there can be little doubt. Just what it is in certain cases of mouth infection it is impossible to say, in the light of present knowledge. There are cases on record which would indicate direct relationship.

ARTERIO-SCLEROSIS, ANEMIA, ETC.

That there is a relation between focal infections, and arterio-sclerosis, anemia, and under certain conditions, iritis, is probable. There is good clinical evidence to support this. Moreover, Rosenow has called attention to the prejudicial effects produced by bacteria upon capillaries adjacent to infected joints as observed by him in his investigations.*

Attention has thus been called to a few of the more common mouth infections, and their systemic effect for the purpose of emphasizing the fact that no physical examination is complete which does not include the mouth; that in it more often than in any other part of the body are to be found chronic foci of infection, which may seriously prejudice the general health: that when present, steps should at once be taken to eliminate them as the only reasonable course to pursue, and to urge in the interest of humanity a closer co-operation between physician and dentist.

59 West Forty-sixth Street.

---

The American Congress on Internal Medicine will hold its first session on December 28-29, 1916, at Hotel Astor, New York City. The program is interesting and gives promise of an interesting and helpful meeting. Any physician interested in this work would do well to get in touch with Dr. Heinrich Stern, secretary general, 250 West Seventy-third Street, New York.

---

Mr. Louis R. Curtis, formerly superintendent of St. Luke's Hospital of Chicago, has been recently elected president of the Frank S. Betz Co., of Hammond, Ind. Mr. Curtis will no doubt prove a very valuable addition to this well known popular firm.

---

Dr. J. M. Martin, of Dallas, Texas, has recently added 25 milligrams more of pure radium element.

---

Do you know that heart disease, pneumonia and tuberculosis cause more than 30 per cent of deaths?

*In an article entitled "The Etiology of Iritis," *Journal A. M. A.*, June 10, 1916, Irions and Brown report one hundred cases of iritis studied by them, eighteen of which were caused by mouth infections, notably dental abscesses.

